# Three Years' Training Adopted

**Published:** 1919-12-06

**Authors:** 


### NURSE TRAINING AT THE LONDON HOSPITAL.

### Three Years' Training Adopted.

### VISCOUNT KNUTSFORD'S STATEMENT TO THE GOVERNORS.

SPEAKING at the Quarterly Court on Wednesday, Lord Knutsford said: We are very anxious to reduce the hours of the nurses' work, and Miss Monk, with our cordial approval, is making out a scheme by which something towards this end can be done. A forty-eight hours week for nurses is impossible. It would be bad for patients to have so constant a change, and the public must remember that if forty-eight hours is the working week of nurses in hospital, then forty-eight hours will be the working week of all nurses. Just think what this means. Patients will need to engage three nurses instead of two. Very few can afford this, and even if they could afford it most people would find it very difficult, if not impossible, to house three nurses. And think what it would mean for a woman in her confinement to have three nurses instead of one.

Then, again, a forty-eight hours week would mean in this hospital an addition of nearly 300 nurses to our staff. Where could they be housed, and how could they be paid? Certainly this hospital cannot afford it, and I do not think any hospitals could. But, setting aside the idea of a forty-eight hours as impracticable, we think we could do something towards reducing the hours by giving more time off and privileges, such as sleeping away from the hospital on their days off, and it is in this direction that our Matron is working hard to see what she can do. But this shortening of hours will involve a great change at this hospital. Up to now we have been able to train a probationer to be a fully qualified nurse in two years. We have always had here the great advantage of a preliminary school for nurses, where our probationers go for seven weeks before they come up to the hospital, and they learn there what would take them much longer if they had to acquire the same knowledge in the busy life of a hospital ward. Then our training has been so organised that after the seven weeks of preliminary training a probationer, during the next two years, is moved about from ward to ward, so that by the end of that time she has had experience of every sort of nursing—women, and men, and children, surgical and medical; and during the two years we have carefully arranged study classes to help them to understand and appreciate and absorb the lectures from the medical staff and from the Matron.

But now, if during these two strenuous years of training we are to shorten the hours, it is obvious we must lengthen the time of training. So we propose to make it three years instead of two. I confess I make this change with great reluctance. I cannot forget that under our system the London Hospital nurses have gained and maintained a wonderfully high reputation. During the war the Head Matron in England, Dame Ethel Becher, was a London Hospital nurse. The Head Matron in France, Dame Maud McCarthy, was a London Hospital nurse; and everywhere, all over the country, London Hospital nurses are holding most important positions and reflecting credit to the training and to the ideals of that great woman, Miss Luckes, who has so lately died. More than this, in addition to those London Hospital nurses who hold these high places, we have for many years past been

**Nurse Training at the London Hospital**—(*continued*).

sending out nurses to private people, and to the most important people in our country, including the late King Edward—and the records of work that they have brought back have been simply astonishingly good.

Just think of this. I have been lately looking through the reports our nurses have brought back from the doctors and patients who have employed them. I have taken the last 1,563 reports we have received, and of these—it is almost unbelievable—we have only nine unsatisfactory reports—nine out of 1,563 reports! and when you remember the different idiosyncrasies of doctors and patients whom these nurses have to satisfy, I say that the result is simply astounding. We have in the office 12,000 such reports, and if this average is kept up—and there is no reason why it should not be—it would mean that out of 12,000 people only seventy-two had anything to say of our nurses except the most extravagant praise and deeply felt gratitude. A marvellous testimony to that great woman's work who was responsible for their training, and who was so well able to inspire them all with her own high ideals.

Naturally, then, I hesitate to propose any change, and I only do so because, as I have said, if the hours are to be shortened in the two years of training, we cannot teach them all she wished to teach them, and all our present Matron wishes to teach them. This is the only reason which makes us propose this change. It has not been forced upon us by any pressure from outside, and if we were not shortening the hours we should not dream of altering a system which has succeeded so wonderfully.

